# Cystinuria in children: diagnosis and treatment

**DOI:** 10.1007/s00345-025-05604-6

**Published:** 2025-04-15

**Authors:** Mehmet İlker Gökçe, Murat Can Karaburun

**Affiliations:** 1 Department of Urology, Ankara University Faculty of Medicine, Altındağ, 06230 Ankara, Turkey; 2 Department of Urology, Etlik City Hospital, Ankara, Turkey

**Keywords:** Cystinuria, Pediatric stones, Urolithiasis, Cystine, Hereditary

## Abstract

**Purpose:**

Cystinuria is the predominant hereditary factor leading to kidney stone formation in the pediatric population. The aim of this manuscript is to provide an overview of cystinuria in children.

**Methods:**

The authors performed a literature review on studies regarding cystinuria in children. A narrative synthesis for analysis of the studies was used.

**Results:**

Cystine is a homodimeric amino acid formed by the disulfide bonding of two cysteine molecules. The problem with this autosomal recessive condition arises from a malfunction in the process of reabsorption. Cystine filtered from the renal glomerulus cannot be reabsorbed from the proximal tubules. Therefore, due to its extremely low solubility at normal urine pH, it precipitates and causes stone formation. Recurrent stone formation is the most prominent clinical presentation of cystinuria. The patients usually present with a renal colic episode with concomitant nausea and hematuria. The aim of medical treatment is to maintain the solubility of cystine in urine. The main strategies are to increase urine volume and urinary pH. Potassium citrate or potassium bicarbonate can be used to raise the pH of the urine to 7.5 to increase cystine solubility. If the treatment with alkalinization and higher urine output fails, cystine binding agents such as tiopronin and D-penicillamine can be added to the treatment. Surgical management of pediatric patients with cystine stones is similar to that in the adult population. However, cystine stones can be resistant to ESWL. Retrograde ureteroscopy with semirigid and flexible instruments is a good option for ureteral stones and also for renal stones less than 20 mm in diameter. The golden standard option for high-volume stones larger than 20 mm in diameter is percutaneous nephrolithotomy (PCNL).

**Conclusions:**

Cystinuria is the primary hereditary factor contributing to the formation of kidney stones throughout childhood. It is a genetic disorder that typically manifests as recurrent stone formations. The aim of the treatment of genetically caused pediatric stone diseases is to prevent stone formation with medical treatments, remove existing stones through surgical treatments, and mitigate the risk of developing chronic kidney disease in the future.

## Introduction

Cystinuria is the most common genetic cause of nephrolithiasis in the pediatric population [1]. Cystinuria is responsible for 4–5% of all incidents of urolithiasis in children [2]. It is an autosomal recessive disorder with an overall prevalence of 1/7000 neonates, and it causes impaired reabsorption of cystine in the proximal tubfules, resulting in stone formation. [3]

There is a significant variation in the clinical presentation of cystinuria, and patients with cystine stones have been shown to have lower health-related quality of life compared to non-cystine stone formers [4]. Additionally, patients with cystinuria were shown to have a high risk of recurrent surgical procedures for stones, and this also increases the risk of chronic kidney disease, renal insufficiency, and therefore, end-stage renal disease. [5]

## Pathophysiology and genetics of cystinuria

Cystine is a homodimeric amino acid formed by the disulfide bonding of two cysteine molecules. In a healthy human kidney, cystine is filtered through the renal glomerulus and subsequently reabsorbed from the proximal tubules through a transport system. The pathogenic mechanism of cystinuria is the defective transepithelial transport of cystine and the dibasic amino acids lysine, arginine, and ornithine in the proximal tubule [6]. In homozygous patients, cystine stone formation is the consequence of the extremely low solubility of cystine in the urine. At normal urine pH, cystine precipitates in the urine and induces the formation of stones [7]. However, urinary stones are not formed by lysine, arginine, or ornithine, as they are significantly more soluble than cystine.

The problem in cystinuria is caused by the impaired production of one of two proteins that play a role in the reabsorption process. Heteromeric amino acid transporters (HATs) involved in cystinuria consist of a heavy subunit (SLC3; HSHAT) and a light subunit (SLC7; LSHAT) connected by a conserved disulfide bridge [8–10]. The heavy subunit seems to be important to transport the carrier to the membrane, while the light subunit provides the transporter function [10, 11]. There are two different genes that are responsible for cystinuria: SLC3 A1 and SLC7 A9. The SLC3 A1 gene encodes for the heavy chain subunit rBAT, and it is located on chromosome 2p16.3-p21, while the SLC7 A9 encodes for the transporting unit of the heterodimeric complex, and it is located on chromosome 19q12 - 13.1 [12, 13]. Patients are classified as either type A or type B. Type A patients have biallelic mutations in SLC3 A1, while type B cystinuria refers to patients who have biallelic SLC7 A9 mutations. If both SLC3 A1 and SLC7 A9 mutations are present, it is classified as type AB [14]. Deletions and duplications in the SLC7 A9 and SLC3 A1 genes are mainly responsible for cystinuria. However, the affected genes or exact genetic mutations cannot be identified in about 10% of the cases [15]. The unexplained cases may result from mutations outside the open reading frame of the SLC7 A9 and SLC3 A1 genes (intronic or promoter regions) or due to mutations in unidentified genes [6]. There may be mixed genotype groups such as AB, where individuals have one pathogenic variant in SLC3 A1 and one in SLC7 A9, and AAB and BBA if there are more than two pathogenic variants pertaining to different genes [16]. The International Cystinuria Consortium (ICC) has reported that type A, type B, and type AB account for 38%, 47%, and 14% of cystinuria patients in their registry. [8, 14]

Of the children with type A cystinuria, homozygous individuals (AA) exhibit urine cystine excretion levels between 2000 and 5000 μmol/gCr, whereas heterozygous individuals (A0) have excretion levels ranging from 25 to 125 μmol/gCr. In type B, homozygous children (BB) exhibit urine cystine excretion comparable to that of type A. Conversely, heterozygous children (B0) demonstrate cystine excretion levels between 100 and 1000 μmol/gCr. The excretion is diminished in the mixed form, type AB, compared to homozygous children (AA or BB). [17]

The excreted amount of cystine and the pH value of the urine are the two important determinants of cystine stone formation. Healthy individuals excrete less than 30 mg of cystine per 1.73 m^2^ of body surface area daily; however, patients homozygous for cystinuria frequently exhibit excretion rates exceeding 400 mg/1.73 m^2^/day. Cystine solubility in urine is roughly 250 mg/L at pH 7.0 and increases significantly with higher pH, reaching 500 mg/L or more above pH 7.5. [18, 19]

## Clinical presentation of cystinuria and diagnosis

Recurrent stone formation is the most prominent clinical presentation of cystinuria. The patients usually present with a renal colic episode with concomitant nausea and hematuria. The episode may result in spontaneous stone passage, but some patients present with a need for intervention. The initial age of stone detection is in the second decade of life in ¾ of the patients [20]. When the pediatric patient presents with a huge or staghorn stone, a family history, and bilateral stones, cystinuria should be investigated. [21]

As cystinuria is a highly recurrent condition, the patients are at risk of chronic kidney disease and end-stage renal failure. Not only the obstruction caused by the stones but also the multiple surgical procedures are responsible for this outcome. Patients with cystinuria were also shown to have a higher risk for this renal failure compared to the calcium oxalate stone patients as well [5, 21]. In a multi-center study investigating the data of 442 patients from 47 different centers, only 22.5% had an eGFR ≥ 90 ml/min per 1.73 m^2^ and 26.7% had an eGFR < 60 ml/min per 1.73 m^2^. A unilateral nephrectomy was reported in 31 of the patients. In this cohort, 28.6% of the patients had hypertension, and this was more prominent in males, older patients, and patients with an eGFR < 60 ml/min per 1.73 m [2]. [22] In another study comparing cystinuria patients and calcium oxalate stone formers, the mean creatinine level was reported to be significantly higher in the former group. Additionally, the rate of nephrectomy was 14.1% in the cystinuria group, while it was only 2.9% in the calcium oxalate stone formers [23]. Among the family members with the same mutation, the clinical presentation can vary significantly. This can be explained by epigenetic mechanisms. Cystinuria exhibits significant phenotypic variability, even among families with identical genetic mutations, indicating potential influence from epigenetic mechanisms, modifying genes, and dietary factors.

Cystinuria can be diagnosed by the examination of kidney stones, the detection of cystine crystals in urinary sediment, or the identification of increased excretion of cystine and dibasic amino acids in the urine [19]. The most straightforward diagnostic test is the microscopic analysis of the urinary sediment from a freshly voided morning urine sample [2]. The identification of flat, hexagonal crystals is pathognomonic during microscopic examination of the urine samples. However, the cystine crystals can be identified in about 2/3 of the patients; therefore, the diagnosis of cystinuria can be validated by the quantification of increased cystine and dibasic amino acids (ornithine, lysine, and arginine) in a 24-h urine collection [19]. While the reference value for cystine excretion in a 24-h urine sample is < 30 mg/d, the expected values for Cystinuria AA/BB genotypes, Cystinuria A0 genotype, and Cystinuria B0 genotype are > 400 mg/d, < 100 mg/d, and 40–400 mg/d, respectively(Table 1) [19, 20]. In a smaller pediatric population, who are unable to manage their voiding, a 24-h urine collection may not be possible, and urinary cystine concentration can be used as a diagnostic method in this group [24]. The first or subsequent morning spot urine specimens are optimal, and findings should be conveyed in relation to the creatinine level (amico acid/creatinine ratio) to adjust for urine concentration [19, 25, 26]. The reference values for urinary cystine concentration in a spot urine sample are < 80 mg/g creatinine for infants younger than 1 month, < 52 mg/g creatinine for infants younger than 1 year, and < 35 mg/g creatinine for children older than 1 year. In children with AA or BB genotype cystinuria, the urinary cystine concentration in a spot urine sample is > 315 mg/g creatinine (Table 2) [17, 19, 25–27]. In pediatric patients evaluated for cystinuria, other conditions with increased urinary cystine excretion, such as tubular immaturity, generalized aminoaciduria (Fanconi syndrome), and organic acidemias, should be considered in the differential diagnosis [2, 28]. The cyanide-nitroprusside test (CNT) is a quick qualitative assay suggested as a screening approach for cystinuria. Nevertheless, owing to its limited reproducibility and sensitivity, as well as the use of toxic and unstable chemicals, this test is often regarded as antiquated [2].

**Table 1:** 24-h urine cystine values

	24-h urine cystine excretion
Normal values	< 30 mg/d
AA and BB genotypes	> 400 mg/d
A0 genotype	< 100 mg/d
B0 genotype	40–400 mg/d

**Table 2:** Spot urine cystine values

	Spot urine cystine concentration
Normal Values for infants < 1 month*	< 80 mg/g creatinine
Normal Values for infants < 1 year*	< 52 mg/g creatinine
Normal Values for children > 1 year*	< 35 mg/g creatinine
Children with AA or BB genotype cystinuria	> 315 mg/g creatinine

*References for the normal values: Parvy PR, Bardet JI, Rabier DM, Kamoun PP. Age-related reference values for free amino acids in first morning urine specimens. Clin Chem. 1988;34(10):2092–2095 [25]., Venta R. Year-long validation study and reference values for urinary amino acids using a reversed-phase HPLC method. Clin Chem. 2001;47(3):575–583 [26]., Tsai MY, Marshall JG, Josephson MW. Free amino acid analysis of untimed and 24-h urine samples compared. Clin Chem. 1980;26(13):1804–1808 [27]. and Kher K, Schnaper HW, Greenbaum LA. Clinical Pediatric Nephrology, Third Edition. CRC Press; 2016

Genetic testing for the mutations is not mandatory for the diagnosis in daily practice. It is rather reserved for research purposes and cases of atypical presentation [19]. Additionally, it is beneficial in the context of genetic counseling for other family members. Given the hereditary nature of cystinuria and the possibility of patients having no symptoms, it is advisable to examine siblings of individuals with cystinuria for the presence of the disease [29]. In a certain group of patients exhibiting prenatal hyper-echogenic colon, genetic testing may be considered for the early detection of cystinuria, since urine amino acid excretion during the first months of life is challenging because of tubular immaturity [19].

Stone formation is the main clinical presentation of cystinuria, and it depends on the urine pH, urine volume, and acid load of the urine. For the imaging of stones, ultrasonography is the initial imaging modality in children, but a low-dose non-contrast CT scan is required if a surgical intervention is planned. Cystine stones show poor radiopacity on x-ray and demonstrate high density on non-contrast CT. Ultrasonography is the ideal imaging modality during the follow-up of the patients, and it can also be advised for the siblings of the patient [16].

Cystinuria is usually diagnosed in the clinical presentation with stone formation. Most of the patients have a family history, and during the surgical procedure with the fragmentation of the stone with laser energy, a characteristic odor presents. Together with that, bubbles form during lithotripsy, and the stones are usually brownish-yellow in color and have a granular surface. The definitive diagnosis after surgery relies on stone analysis with the x-ray diffraction method.

## Medical treatment

The aim of medical treatment is to maintain the solubility of cystine in urine. To achieve this aim, the main strategies are to increase urine volume and urinary pH [16]. The desired urine output in children is 2 L/1.73 m2 per day [2, 16, 19, 30] and in the adult population, 2.5–3 L per day is the minimum desired urine output [5]. Particular attention must be placed on nocturnal hours, as urine can attain a supersaturated state with cystine. Consequently, patients have to be instructed to consume fluids before bedtime and upon awakening [17].

Dietary management for patients with cystinuria includes recommendations for a low daily sodium intake and low methionine-rich content. These dietary interventions may decrease cystine synthesis and excretion. Decreased methionine consumption diminishes cystine synthesis, yet methionine intake shouldn’t fall below the physiological need [16, 21]. Avoidance of a methionine-rich diet can reduce the acid load of the urine as well, and this contributes to alkalinization [31]. However, adhering to a methionine-restricted diet is very challenging, given that the majority of foods contain the amino acid methionine [32]. Especially in the pediatric population, a general protein restriction should not be recommended since it may impede their growth. The urine excretion of cystine and dibasic amino acids in individuals with cystinuria is correlated with urinary sodium excretion [33]. Therefore, dietary sodium restriction is advised for the management of cystinuria [6, 34]. For the pediatric population, sodium intake of less than 1.5 mEq/kg/day is advised. With a low-sodium diet, an increased intake of fruit juice, mostly citrus, can be recommended. The citrus includes citric acid and potassium, hence elevating both diuresis and alkali burden [17].

In order to enhance the solubility of cystine, it is necessary to raise the pH of urine to a level of 7.5. For oral alkalinization, potassium citrate or potassium bicarbonate can be used [2, 35]. Potassium citrate has been shown to provide sufficient urine alkalization with the proper dose [36, 37]. In pediatric patients, potassium citrate at a dosage of 60–80 mEq/1.73 m [2]/d is recommended as an appropriate alkalinizing agent due to its efficacy in alkalinization without elevating sodium excretion [37, 38]. The daily dose in children can be calculated based on body surface area or total body weight [39]. In pediatric patients, potassium citrate therapy starts with the minimum dosage and requires routine urine pH assessment three times or more daily [36]. The recommended starting dose for potassium citrate is 0.5–1.0 mEq/kg/d and is recommended to be given at least 2–3 times daily [16, 39, 40]. For example, the starting dose for a 30 kg child is 30 mEq per day and can be given in 3 separate doses of 10 mEq each time. Routinely, potassium citrate can be administered in 3–4 separate doses or diluted in a substantial amount of water [19]. The dosage may be adjusted based on urine pH monitoring, up to a maximum of 3–4 mEq per kilogram per day [17]. It is also important to take the last dose of the medication just before going to bed, which will provide alkaline pH until the next dose in the morning [17]. However, it is not always straightforward for the child and family to adhere to this dose regimen and maintain sufficient fluid consumption [41, 42]. This poses a challenge, especially for younger pediatric patients. In very young children or older children incapable of adhering to the prescribed medical treatment and fluid intake, the dosage regimen for citrate medications and fluid intake can be attained by the use of alternate enteral feeding systems, such as nasogastric or gastrostomy tubes [43]. One other agent that can be used for alkalinization, sodium bicarbonate, is primarily indicated for patients with severe renal insufficiency or those who cannot tolerate potassium citrate, despite the fact that sodium may elevate urinary cystine excretion. [19, 36]

If the treatment with alkalinization and higher urine output fails, cystine binding agents can be added to the treatment. Tiopronin (α-mercaptoproprionyl-glycine) and D-penicillamine can bind cystine to form cysteine-tiopronin and cysteine-penicillamine complexes, respectively. By this way, free cystine levels are reduced. In comparison to the solubility of cystine, the solubility of the penicillamine-cysteine complex is up to fifty times higher [6]. In pediatric patients, the dosing of tiopronin starts with 15 mg/kg per day in three divided doses [17]. The dosage for tiopronin can be adjusted as necessary to keep urine cystine < 300 mg/L [2]. Tiopronin medication can be dosed according to age and body weight and is primarily given 1–2 times a day in patients < 2 years of age, between 2–3 times a day in those being 3–6, 2 times a day in patients from 6 to 9 and then three times a day in the older patients. For penicillamine, the dosage in children is 20 to 30 mg/kg per day (maximum 1.2 g/day) in three to four divided doses [19, 36, 44]. Although both tiopronin and D-penicillamine effectively decrease urine cystine excretion, regular use may not be possible for all patients due to significant adverse effects. Although older studies indicate that tiopronin has a less serious adverse effect profile, more current research suggests that both medications have comparable adverse effect profiles [2, 19, 35, 36]. Taste disturbances, mucocutaneous lesions, immune-mediated reactions, and various skin rashes may be observed in relation to the medications [22]. Neutropenia and thrombocytopenia have rarely been reported among hematologic side effects [45]. Proteinuria may develop due to these drugs, and nephrotic syndrome may develop due to immune complex membranous glomerulopathy in long-term use. Proteinuria usually occurs after several months of treatment. If nephrotic syndrome develops, the drug should be discontinued [46, 47]. Deficiencies in zinc and copper may arise from chelation, necessitating supplementary intake. Due to penicillamine's antagonistic effect on vitamin B6 action, a supplementation of pyridoxine (vitamin B6) is advised during therapy [45, 48]. Consequently, these patients should be routinely checked for adverse effects [36, 49]. The duration of treatment should be adjusted on a patient-specific basis, taking into account the patient’s clinical status and potential adverse effects.

The objective of pharmacological treatment using cystine-binding thiol agents is to maintain low urine cystine concentrations while minimizing adverse effects of the medicine. Therefore, it is important to monitor urinary cystine levels. Techniques that differentiate cystine from drug–cysteine complexes require complex disruption, complicating efficacy assessment. A solid-phase assay provides a reliable method to evaluate urinary cystine saturation and capacity in the presence of these medications [34, 50]. In this manner, necessary dosage modifications can be implemented, and cystine concentrations can be maintained at optimal levels.

The angiotensin-converting enzyme inhibitor captopril and meso- 1,3-dimercaptosuccinic acid are sulfhydryl compounds that can form highly soluble disulfides with cysteine. However, the results of studies examining the efficacy of these agents in cystinuria are inconsistent [51–53]. Thus, these medicines are not advised as treatment options for cystinuria.

Recent studies have focused on L-cystine diamides as a new therapeutic option to inhibit L-cystine crystallization in the management of cystinuria. While several studies have shown their efficacy in reducing cystine stone formation, current evidence for their therapeutic use remains insufficient [54, 55]. Another agent under investigation, α-Lipoic acid, has been shown to inhibit cystine stone formation in mice [56]. Although it was reported to decrease cystine supersaturation and increase cystine solubility in the series published as a case report, a higher level of evidence is needed to make clearer inferences and recommendations. [57]

Sibnayal**®** (a combination of potassium citrate and potassium hydrogen carbonate), a new alkalinizing agent with a prolonged-release formulation, has been approved and introduced in distal renal tubular acidosis [58]. The primary benefits of this medication, shown to be efficacious in the distal RTA group vs. conventional alkaline treatments, are its minimal adverse effect profile and low number of daily doses [59]. Although no proof currently exists within the cystinuria group, in the future, its use may enhance quality of life and treatment adherence in this population with its advantages.

Patient compliance with medical treatment and follow-up is an essential component in the prevention of stone recurrence. Patients who had a high level of compliance with postoperative medical treatment and follow-up regimens were shown to have a lower incidence of stone recurrence. Additionally, patients who were non-compliant with medical treatment following the initial intervention had a shorter time to recurrence [42]. Thus, patients must be pushed to adhere to their follow-up schedules.

## Surgical treatment

Observation is an effective strategy for managing small asymptomatic renal stones; however, the emergence of stone growth, pain, infection, or hematuria usually requires surgical intervention. In pediatric patients, it is crucial to assess the risks associated with both intervention and non-intervention, including the potential for loss of kidney function, hypertension, and chronic renal failure. In cases of stone growth, pain, infection, hematuria, or obstruction, timely surgical intervention is essential to mitigate these risks.

Surgical treatment of pediatric patients with cystine stones is not different from the adult population. Extracorporeal Shock Wave Lithotripsy (ESWL) can be considered an option for pediatric patients when the highly recurrent nature of the disease is considered. However, it should be kept in mind that cystine stones can be resistant to ESWL, and the pediatric patient should be anesthetized for ESWL [60]. Retrograde ureteroscopy with semirigid and flexible instruments is a good option for ureteral stones, as well as for renal stones less than 20 mm in diameter. The golden standard option for high-volume stones larger than 20 mm in diameter is percutaneous nephrolithotomy (PCNL) [61]. Miniaturized PCNL has the potential to diminish the amount of renal trauma related to surgery and also makes it possible for the patients to be discharged without any tubes. Staged ureteroscopy is also an option for cases where PNL is not available; staged URS (i.e., performed in separate, planned sessions) is an alternative option for larger stones.

Given the high recurrence rate in cystinuric children, endoscopic treatments such as RIRS or PCNL should aim to remove all stone fragments, as even millimetric residual stones might lead to regrowth and aggregation. Ultimately, despite an apparently favorable surgical result, aggressive continuation of medical therapy may be necessary [62, 63]. Patients with cystinuria who are stone-free, when managed under the SaFER (stone and fragments fully removed) protocol, need fewer further procedures compared to those who are not stone-free [64]. Additionally, multiple surgical procedures (mostly > 3) were linked to worse renal function over time compared to those with fewer operations [62]. In summary, the management of pediatric cystine stones should focus on the complete surgical removal of the stone, the prevention of recurrence with general precautions and pharmacological intervention, and the maintenance of renal function.

## Follow-up

Considering a higher risk of recurrent surgical interventions and chronic renal failure in patients with cystinuria, their close follow-up is crucial. However, the frequency of follow-up is variable, and there are no clear recommendations regarding this specific patient group [65]. Given that these individuals are classified as high-risk stone formers, the EAU guidelines’ recommendations for this patient group can be accepted. Lifelong follow-up is recommended in this scenario. The recommended frequency of follow-up is biannually in the first year post-surgical intervention, followed by annually thereafter [65]. Personalized follow-up protocols may be implemented on an individual patient basis, ranging from every three months to annualy. [66]

Follow-up should include imaging, metabolic evaluations, and, if applicable, an assessment of medication adherence and any adverse effects related to the medications [65]. Ultrasound should be used as the primary imaging technique in the follow-up of these patients [65]. Estimated GFR, serum full blood and biochemistry analysis, urinary protein excretion, and urine pH should be assessed.

## Data Availability

No datasets were generated or analysed during the current study.

## References

[1] D’Ambrosio V, Capolongo G, Goldfarb D, Gambaro G, Ferraro PM (2022) Cystinuria: an update on pathophysiology, genetics, and clinical management. Pediatr Nephrol 37(8):1705–1711 34812923 10.1007/s00467-021-05342-y

[2] Claes DJ, Jackson E (2012) Cystinuria: mechanisms and management. Pediatr Nephrol 27(11):2031–2038 22281707 10.1007/s00467-011-2092-6

[3] Eggermann T, Venghaus A, Zerres K (2012) Cystinuria: an inborn cause of urolithiasis. Orphanet J Rare Dis 7:19 22480232 10.1186/1750-1172-7-19 PMC3464901

[4] Streeper NM, Wertheim ML, Nakada SY, Penniston KL (2017) Cystine stone formers have impaired health-related quality of life compared with noncystine stone formers: a case-referent study piloting the wisconsin stone quality of life questionnaire among patients with cystine stones. J Endourol 31(S1):S48–S53 27717296 10.1089/end.2016.0564

[5] Barbey F, Joly D, Rieu P, Méjean A, Daudon M, Jungers P (2000) Medical treatment of cystinuria: critical reappraisal of long-term results. J Urol 163(5):1419–1423 10751848 10.1016/s0022-5347(05)67633-1

[6] Chillarón J, Font-Llitjós M, Fort J et al (2010) Pathophysiology and treatment of cystinuria. Nat Rev Nephrol 6(7):424–434 20517292 10.1038/nrneph.2010.69

[7] Mattoo A, Goldfarb DS (2008) Cystinuria. Semin Nephrol 28(2):181–191 18359399 10.1016/j.semnephrol.2008.01.011

[8] Palacín M, Nunes V, Font-Llitjós M et al (2005) The genetics of heteromeric amino acid transporters. Physiology (Bethesda) 20:112–124 15772300 10.1152/physiol.00051.2004

[9] Palacín M, Kanai Y (2004) The ancillary proteins of HATs: SLC3 family of amino acid transporters. Pflugers Arch 447(5):490–494 14770309 10.1007/s00424-003-1062-7

[10] Fotiadis D, Kanai Y, Palacín M (2013) The SLC3 and SLC7 families of amino acid transporters. Mol Aspects Med 34(2–3):139–158 23506863 10.1016/j.mam.2012.10.007

[11] Bröer S, Palacín M (2011) The role of amino acid transporters in inherited and acquired diseases. Biochem J 436(2):193–211 21568940 10.1042/BJ20101912

[12] Feliubadalo L, Font M, Purroy J et al (1999) Non-type I cystinuria caused by mutations in SLC7A9, encoding a subunit (bo,+AT) of rBAT. Nat Genet 23(1):52–57 10471498 10.1038/12652

[13] Calonge MJ, Gasparini P, Chillaron J et al (1994) Cystinuria caused by mutations in rBAT, a gene involved in the transport of cystine. Nat Genet 6(4):420–425 8054986 10.1038/ng0494-420

[14] Font-Llitjós M, Jiménez-Vidal M, Bisceglia L et al (2005) New insights into cystinuria: 40 new mutations, genotype-phenotype correlation, and digenic inheritance causing partial phenotype. J Med Genet 42(1):58–68 15635077 10.1136/jmg.2004.022244 PMC1735913

[15] Parvari R, Hershkovitz E (2007) Chromosomal microdeletions and genes’ functions: a cluster of chromosomal microdeletions and the deleted genes’ functions. Eur J Hum Genet 15(10):997–998 17625506 10.1038/sj.ejhg.5201894

[16] Emma F, Goldstein SL, Bagga A, Bates CM, Shroff R (2022) Pediatric Nephrology. Springer International Publishing

[17] Kher K, Schnaper HW, Greenbaum LA (2016) Clinical pediatric nephrology, 3rd edn. CRC Press

[18] Joly D, Rieu P, Méjean A, Gagnadoux MF, Daudon M, Jungers P (1999) Treatment of cystinuria. Pediatr Nephrol 13(9):945–950 10603157 10.1007/s004670050736

[19] Servais A, Thomas K, Dello Strologo L et al (2021) Cystinuria: clinical practice recommendation. Kidney Int 99(1):48–58 32918941 10.1016/j.kint.2020.06.035

[20] Dello Strologo L, Pras E, Pontesilli C et al (2002) Comparison between SLC3A1 and SLC7A9 cystinuria patients and carriers: a need for a new classification. J Am Soc Nephrol 13(10):2547–2553 12239244 10.1097/01.asn.0000029586.17680.e5

[21] Rhodes HL, Yarram-Smith L, Rice SJ et al (2015) Clinical and genetic analysis of patients with cystinuria in the United Kingdom. Clin J Am Soc Nephrol 10(7):1235–1245 25964309 10.2215/CJN.10981114 PMC4491297

[22] Prot-Bertoye C, Lebbah S, Daudon M et al (2015) CKD and its risk factors among patients with cystinuria. Clin J Am Soc Nephrol 10(5):842–851 25717071 10.2215/CJN.06680714 PMC4422236

[23] Assimos DG, Leslie SW, Ng C, Streem SB, Hart LJ (2002) The impact of cystinuria on renal function. J Urol 168(1):27–30 12050485

[24] Wong KA, Pardy C, Pillay S et al (2016) Can the presence of crystalluria predict stone formation in patients with cystinuria? J Endourol 30(5):609–614 26781171 10.1089/end.2015.0692

[25] Parvy PR, Bardet JI, Rabier DM, Kamoun PP (1988) Age-related reference values for free amino acids in first morning urine specimens. Clin Chem 34(10):2092–2095 3168222

[26] Venta R (2001) Year-long validation study and reference values for urinary amino acids using a reversed-phase HPLC method. Clin Chem 47(3):575–583 11238314

[27] Tsai MY, Marshall JG, Josephson MW (1980) Free amino acid analysis of untimed and 24-h urine samples compared. Clin Chem 26(13):1804–1808 7192188

[28] Milliner DS (1990) Cystinuria. Endocrinol Metab Clin North Am 19(4):889–907 2081517

[29] Ferraro PM, D’Addessi A, Gambaro G (2013) When to suspect a genetic disorder in a patient with renal stones, and why. Nephrol Dial Transplant 28(4):811–820 23291371 10.1093/ndt/gfs545

[30] Geary DF, Schaefer F (2008) Comprehensive pediatric nephrology e-book: text with CD-ROM. Elsevier Health Sciences

[31] Norman RW, Manette WA (1990) Dietary restriction of sodium as a means of reducing urinary cystine. J Urol 143(6):1193–1195 2342181 10.1016/s0022-5347(17)40222-9

[32] Kolb FO, Earll JM, Harper HA (1967) “Disappearance” of cystinuria in a patient treated with prolonged low methionine diet. Metabolism 16(4):378–381 6024162 10.1016/0026-0495(67)90049-2

[33] Jaeger P, Portmann L, Saunders A, Rosenberg LE, Thier SO (1986) Anticystinuric effects of glutamine and of dietary sodium restriction. N Engl J Med 315(18):1120–1123 3093863 10.1056/NEJM198610303151803

[34] Goldfarb DS, Coe FL, Asplin JR (2006) Urinary cystine excretion and capacity in patients with cystinuria. Kidney Int 69(6):1041–1047 16501494 10.1038/sj.ki.5000104

[35] Prot-Bertoye C, Lebbah S, Daudon M et al (2019) Adverse events associated with currently used medical treatments for cystinuria and treatment goals: results from a series of 442 patients in France. BJU Int 124(5):849–861 30801923 10.1111/bju.14721

[36] Knoll T, Zöllner A, Wendt-Nordahl G, Michel MS, Alken P (2005) Cystinuria in childhood and adolescence: recommendations for diagnosis, treatment, and follow-up. Pediatr Nephrol 20(1):19–24 15602663 10.1007/s00467-004-1663-1

[37] Fjellstedt E, Denneberg T, Jeppsson JO, Tiselius HG (2001) A comparison of the effects of potassium citrate and sodium bicarbonate in the alkalinization of urine in homozygous cystinuria. Urol Res 29(5):295–302 11762790 10.1007/s002400100200

[38] Sakhaee K, Nicar M, Hill K, Pak CY (1983) Contrasting effects of potassium citrate and sodium citrate therapies on urinary chemistries and crystallization of stone-forming salts. Kidney Int 24(3):348–352 6645208 10.1038/ki.1983.165

[39] Rees L, Bockenhauer D, Webb NJA, Punaro MG (2019) Paediatric Nephrology. OUP Oxford

[40] Tekin A, Tekgul S, Atsu N, Sahin A, Bakkaloglu M (2001) Cystine calculi in children: the results of a metabolic evaluation and response to medical therapy. J Urol 165(6 Pt 2):2328–2330 11371943 10.1016/S0022-5347(05)66196-4

[41] Pietrow P, Auge BK, Weizer AZ et al (2003) Durability of the medical management of cystinuria. J Urol 169(1):68–70 12478105 10.1016/S0022-5347(05)64037-2

[42] Asi T, Dogan HS, Bozaci AC, Citamak B, Altan M, Tekgul S (2020) A single center’s experience in pediatric cystine stone disease management: what changed over time? Urolithiasis 48(6):493–499 32556828 10.1007/s00240-020-01200-y

[43] Kowalczyk NS, Zisman AL (2021) Cystinuria: Review of a Life-long and Frustrating Disease. Yale J Biol Med 94(4):681–686 34970106 PMC8686768

[44] Dello Strologo L, Laurenzi C, Legato A, Pastore A (2007) Cystinuria in children and young adults: success of monitoring free-cystine urine levels. Pediatr Nephrol 22(11):1869–1873 17694338 10.1007/s00467-007-0575-2

[45] Jaffe IA (1986) Adverse effects profile of sulfhydryl compounds in man. Am J Med 80(3):471–476 2937293 10.1016/0002-9343(86)90722-9

[46] Tasic V, Lozanovski VJ, Ristoska-Bojkovska N, Sahpazova E, Gucev Z (2011) Nephrotic syndrome occurring during tiopronin treatment for cystinuria. Eur J Pediatr 170(2):247–249 20924604 10.1007/s00431-010-1315-3

[47] Habib GS, Saliba W, Nashashibi M, Armali Z (2006) Penicillamine and nephrotic syndrome. Eur J Intern Med 17(5):343–348 16864010 10.1016/j.ejim.2006.03.001

[48] Tomono I, Abe M, Matsuda M (1973) Effect of penicillamine (a vitamin B6 antagonist) on pyridoxal enzymes. J Biochem 74(3):587–592 4148378 10.1093/oxfordjournals.jbchem.a130280

[49] Zhong H, Wang L, Gu ZC, Cui M, Liu XY, Pang XY (2019) Minimal change disease induced by tiopronin: a rare case report and a review of the literature. Ann Transl Med 7(16):398 31555712 10.21037/atm.2019.07.42 PMC6736807

[50] Coe FL, Clark C, Parks JH, Asplin JR (2001) Solid phase assay of urine cystine supersaturation in the presence of cystine binding drugs. J Urol 166(2):688–693 11458118

[51] Coulthard MG, Richardson J, Fleetwood A (1995) The treatment of cystinuria with captopril. Am J Kidney Dis 25(4):661–662 7702068 10.1016/0272-6386(95)90142-6

[52] Maiorino RM, Bruce DC, Aposhian HV (1989) Determination and metabolism of dithiol chelating agents. VI. Isolation and identification of the mixed disulfides of meso-2,3-dimercaptosuccinic acid with L-cysteine in human urine. Toxicol Appl Pharmacol 97(2):338–349 2538007 10.1016/0041-008x(89)90338-4

[53] Sloand JA, Izzo JL Jr (1987) Captopril reduces urinary cystine excretion in cystinuria. Arch Intern Med 147(8):1409–1412 2820331

[54] Sahota A, Tischfield JA, Goldfarb DS, Ward MD, Hu L (2019) Cystinuria: genetic aspects, mouse models, and a new approach to therapy. Urolithiasis 47(1):57–66 30515543 10.1007/s00240-018-1101-7 PMC6592844

[55] Yang Y, Albanyan H, Lee S et al (2018) Design, synthesis, and evaluation of l-cystine diamides as l-cystine crystallization inhibitors for cystinuria. Bioorg Med Chem Lett 28(8):1303–1308 29571572 10.1016/j.bmcl.2018.03.024 PMC5893393

[56] Zee T, Bose N, Zee J et al (2017) α-Lipoic acid treatment prevents cystine urolithiasis in a mouse model of cystinuria. Nat Med 23(3):288–290 28165480 10.1038/nm.4280 PMC5656064

[57] Cil O, Perwad F 2020 α-Lipoic acid (ALA) improves cystine solubility in cystinuria: report of 2 cases. Pediatrics, 145(5) 10.1542/peds.2019-2951 32245805

[58] Bertholet-Thomas A, Guittet C, Manso-Silván MA et al (2021) Efficacy and safety of an innovative prolonged-release combination drug in patients with distal renal tubular acidosis: an open-label comparative trial versus standard of care treatments. Pediatr Nephrol 36(1):83–91 32712761 10.1007/s00467-020-04693-2 PMC7701073

[59] Tan HL, Marlais M, Veligratli F, Shah S, Hayes W, Bockenhauer D (2024) Treatment of paediatric renal tubular acidosis with a prolonged-release alkali supplementation. Pediatr Nephrol 39(11):3373–3375 38771324 10.1007/s00467-024-06411-8

[60] Landau EH, Shenfeld OZ, Pode D et al (2009) Extracorporeal shock wave lithotripsy in prepubertal children: 22-year experience at a single institution with a single lithotriptor. J Urol 182(4 Suppl):1835–1839 19692011 10.1016/j.juro.2009.04.084

[61] Cohen J, Cohen S, Grasso M (2013) Ureteropyeloscopic treatment of large, complex intrarenal and proximal ureteral calculi. BJU Int 111(3):E127-131 22757752 10.1111/j.1464-410X.2012.11352.x

[62] Ripa F, Pietropaolo A, Geraghty R, Griffin S, Cook P, Somani B (2023) Outcomes of paediatric cystine stone management: results of a systematic review. Curr Urol Rep 24(8):371–380 37079195 10.1007/s11934-023-01162-9

[63] Tiselius HG (2010) New horizons in the management of patients with cystinuria. Curr Opin Urol 20(2):169–173 19887942 10.1097/MOU.0b013e328333b674

[64] Moore SL, Somani BK, Cook P (2019) Journey of a cystinuric patient with a long-term follow-up from a medical stone clinic: necessity to be SaFER (stone and fragments entirely removed). Urolithiasis 47(2):165–170 29696300 10.1007/s00240-018-1059-5 PMC6420894

[65] Skolarikos A, Neisius A, Petřík A, et al. Urolithiasis. Paper presented at: EAU Guidelines. Edn. presented at the EAU Annual Congress Amsterdam2022

[66] Thomas K, Wong K, Withington J, Bultitude M, Doherty A (2014) Cystinuria-a urologist’s perspective. Nat Rev Urol 11(5):270–277 24662732 10.1038/nrurol.2014.51

